# Development, validation and comparison of multivariable risk scores for prediction of total stroke and stroke types in Chinese adults: a prospective study of 0.5 million adults

**DOI:** 10.1136/svn-2021-001251

**Published:** 2022-03-15

**Authors:** Matthew Chun, Robert Clarke, Tingting Zhu, David Clifton, Derrick A Bennett, Yiping Chen, Yu Guo, Pei Pei, Jun Lv, Canqing Yu, Ling Yang, Liming Li, Zhengming Chen, Benjamin J Cairns, the China Kadoorie Biobank Collaborative Group

**Affiliations:** 1 Clinical Trial Service Unit and Epidemiological Studies, Nuffield Department of Population Health, University of Oxford, Oxford, UK; 2 Department of Engineering Science, University of Oxford, Oxford, UK; 3 Department of Biomedical Engineering, Oxford-Suzhou Centre for Advanced Research, Suzhou, China; 4 Medical Research Council Health Research Unit, Nuffield Department of Population Health, University of Oxford, Oxford, UK; 5 CKB Project Department, Fuwai Hospital Chinese Academy of Medical Sciences, National Center for Cardiovascular Diseases, Beijing, China; 6 CKB Project Department, Chinese Academy of Medical Sciences, Beijing, China; 7 Department of Epidemiology and Biostatistics, School of Public Health, Peking University, Beijing, China; 8 Department of Epidemiology, Peking University Center for Public Health and Epidemic Preparedness and Response, Beijing, China

**Keywords:** stroke, risk factors, prospective studies, standard of care

## Abstract

**Background and purpose:**

Low-income and middle-income countries have the greatest stroke burden, yet remain understudied. This study compared the utility of Framingham versus novel risk scores for prediction of total stroke and stroke types in Chinese adults.

**Methods:**

China Kadoorie Biobank (CKB) is a prospective study of 512 726 adults, aged 30–79 years, recruited from 10 areas in China in 2004–2008. By 1 January 2018, 43 234 incident first stroke cases (36 310 ischaemic stroke (IS); 8865 haemorrhagic stroke (HS)) were recorded in 503 842 participants with no history of stroke at baseline. We compared the predictive utility of the Framingham Stroke Risk Profile (FSRP) with novel CKB stroke risk scores and included recalibration, refitting, stratifying by study area and addition of other risk factors. Discrimination was assessed using area under the receiver operating characteristic curve (AUC) and calibration was assessed using Greenwood-Nam-D’Agostino χ^2^ statistics.

**Results:**

Incidence of total stroke varied fivefold by area in China. The FSRP had good discrimination for total stroke (AUC (95% CI); men: 0.78 (0.77 to 0.79), women: 0.77 (95% CI 0.76 to 0.78)), but poor calibration (χ^2^; men: 1,825, women: 3,053), substantially underestimating absolute risks. Recalibration reduced χ^2^ by >80%, but did not improve discrimination. Refitting the FSRP did not materially improve discrimination, but further improved calibration. Stratification by area improved discrimination (AUC; men: 0.82 (0.82 to 0.83); women: 0.82 (0.82 to 0.83)), but not calibration. Adding other risk factors yielded modest, but statistically significant, improvements in the AUCs. The findings for IS and HS were similar to those for total stroke.

**Conclusions:**

The FSRP reliably differentiated Chinese adults with incident stroke, but substantially underestimated the absolute risks of stroke. Novel local risk prediction equations that took account of differences in stroke incidence within China enhanced risk prediction of total stroke and major stroke pathological types.

## Introduction

Stroke is a leading cause of death and disability worldwide, and about three-quarters of all stroke cases now occur in low-income and middle-income countries (LMICs), including China.^1^ Stroke accounted for 34 million prevalent cases and 2 million deaths in China in 2017.^2^ Cost-effective primary prevention of stroke requires both population-based lifestyle strategies (eg, salt reduction) and blood pressure-lowering and lipid-lowering medication in high-risk individuals.^3^ Risk prediction equations are required to identify those who would derive maximum benefit from such preventive treatments.^4 5^

The Framingham Stroke Risk Profile (FSRP), derived from a multigeneration prospective cohort study in Framingham, Massachusetts, USA, is a widely used risk score for prediction of stroke.^5–8^ It provides sex-specific predictions of the absolute risks of total stroke within a specified interval (typically in the next 10 years), based on age, current smoking, history of coronary heart disease (CHD), atrial fibrillation (AF), diabetes, systolic blood pressure and use of antihypertensive treatment.^6–8^ Recently updated in 2017, the FSRP has been validated in many high-income countries to predict risk of total stroke,^8 9^ but its clinical utility in LMICs, such as China, is uncertain.

The incidence rates of total stroke are higher in China than in Western populations, as are the proportions with haemorrhagic stroke (HS).^10^ Within China there are well-documented large, although unexplained differences in the incidence of stroke between geographical areas.^11^ Previous studies that estimated absolute risk of total stroke in Chinese populations were constrained by insufficient numbers of stroke cases, involvement of single rather than multiple areas, lack of reliable information on stroke types (eg, ischaemic stroke (IS) vs HS) and lack of contemporary evidence.^12–14^ Consequently, there is a need for more reliable prediction of absolute risks of total stroke and stroke types in Chinese individuals to guide targeted use of evidence-based cost-effective treatments including lipid-lowering and antiplatelet therapy.^15^

Using data from a large prospective study of 0.5M adults recruited into the China Kadoorie Biobank (CKB) in 2004–2008, we compared the performance of the established FSRP with newly developed and internally validated local risk equations to predict the absolute risks of total stroke and stroke types in Chinese adults. The aims of the present report were to develop and validate multivariable risk scores for prediction of total stroke, IS and HS in men and women living in China, and to compare the predictive value of (1) the 2017 FSRP; (2) a recalibrated FSRP; (3) a local recalibrated and refitted FSRP; (4) a recalibrated and refitted FSRP after stratifying by geographical area and (5) area-stratified, recalibrated and refitted models with additional risk factors. A risk calculator for total stroke and stroke pathological types is provided to enable other investigators to validate these stroke risk scores in independent populations.

## Methods

### Study population

The data included in the present analyses are available from the corresponding author on reasonable request. Details of the design and methods used in the CKB have been previously reported.^16 17^ Briefly, the CKB is a prospective cohort study of 512 726 participants, aged 30–79 years, enrolled from 10 geographically diverse areas (5 urban, 5 rural) of China in 2004–2008. An interviewer-administered electronic questionnaire was used to collect data on sociodemographic factors, lifestyle factors (eg, smoking, alcohol, diet), medical history and current medication and physical activity. Physical measurements included height, weight, hip and waist circumference, bioimpedance, blood pressure and heart rate. All participants provided a blood sample, and random plasma glucose levels were estimated to screen for diabetes. All participants provided written informed consent.

### Follow-up for stroke outcomes

The vital status of participants was monitored through death registries supplemented by annual checks with local residential records and active confirmation by contacting local street committees or village administrators.^17^ All hospitalised cases of stroke were identified by electronic linkage to established registries of major diseases and health insurance records (covering >97% of participants), supplemented by annual home visits for uninsured participants. All fatal and non-fatal stroke cases were coded by trained medical staff using the International Classification of Diseases 10th revision. The major pathological types of stroke were IS (I63), HS (I60 and I61) and unspecified stroke (I64) (online supplemental eMethods II).^18^

10.1136/svn-2021-001251.supp1 Supplementary data

### Statistical analyses

The present analyses were restricted to individuals with no prior history of stroke or transient ischaemic attack (205 293 men, 298 549 women) at the date of recruitment. The participants were followed up to detect stroke and death until 1 January 2018, and all incident cases of first stroke (19 587 strokes in men; 23 647 strokes in women) that were recorded for up to 9 years after the baseline survey were included.

For consistency with the sex-specific FSRP and current clinical practice, the present analyses were performed separately in men and women, using time-in-study as the time scale of interest. First, CKB individuals were randomly divided into a training set (85%) and test set (15%). The FSRP was then applied in the test set to predict the risk of total stroke for each individual within 9 years of the baseline survey. Since AF was not recorded in CKB, the FSRP predictions were calculated assuming AF was absent at baseline. No major violations of the proportional hazards assumption for the traditional FSRP covariates were identified (online supplemental eMethods III).

A recalibrated model (‘+Recalibration’) was subsequently developed, using the Breslow estimator to derive a baseline survival function that adjusted for the mean values of risk factors in CKB,^19 20^ while retaining the 2017 FSRP HRs.^8^ A recalibrated and refitted model (‘+Refitting’) was then constructed using Cox regression to derive new HRs for the FSRP risk factors in CKB. For recalibration and refitting, model parameters were derived from the training set, and all models were evaluated using the test set.

To adjust for differences in baseline hazards across the 10 CKB areas, we next developed a model (‘+Area stratification’) with separate area-specific baselines estimated at the sex-specific mean risk factor values for the overall CKB. In this model, area-stratified Cox regression was used to estimate new HRs for the FSRP risk factors. The model was constructed from the training set and evaluated in the test set.

After estimating separate area-specific baselines, we finally developed an expanded model (‘+Additional risk factors’) using 133 additional risk indicators recorded at baseline in CKB (online supplemental eWorkbook I), including sociodemographic factors, diet, alcohol consumption, personal and family medical history, physical activity, and physical measurements.^17^ The 133 additional risk factors were selected based on their suspected relationship with stroke, while excluding laboratory-based tests, genetic information, and brain imaging that are not widely available in lower-resource clinical settings in China. A subset of these risk factors was then selected automatically using 10-fold cross-validated, least absolute shrinkage and selection operator (LASSO) regularisation (a technique that penalises the inclusion of additional risk factors to prevent overfitting) within the training set,^21 22^ and the selected risk factors were used to fit an area-stratified Cox model using the complete training set. Evaluation of the fitted model was performed using the test set.

10.1136/svn-2021-001251.supp2 Supplementary data

Since the associations of individual risk factors with stroke pathological types differ,^15 18 23–25^ we also hypothesised that developing separate risk equations for IS and HS could further improve predictive performance compared with a single model for total stroke. Hence, we repeated the analyses separately for IS and for HS pathological types.

To compare predictive performance across models, each model was assessed for discrimination and calibration of 9-year stroke risk predictions using the test set. Risk discrimination refers to the ability to correctly discriminate between individuals with and without stroke, and was evaluated using the area under the receiver operating characteristic curve (AUC). The AUCs for each model were compared with the FSRP using Delong’s test.^26^ Calibration refers to the similarity between observed and predicted absolute risks and was evaluated using calibration plots. The Greenwood-Nam-D’Agostino χ^2^ test statistic was used to compare the observed incidence (calculated as 1 − Kaplan-Meier survival probability) and predicted risks by deciles of predicted risk (with lower χ^2^ values indicating better model calibration).^27 28^ The 95% CIs were constructed for AUCs using 1000 bootstrapped samples from the test set. For models with area-specific baselines, AUCs were evaluated for both the overall study population and separately within each CKB area.

Sensitivity analyses included restricting the age range to those ≥55 years (for fair comparison with the Framingham study), adding risk factors to the FSRP prior to stratification by study area (to assess the reordering of incremental modelling improvements), and implementing cumulative incidence functions and Fine-Gray models (to account for the competing risk of death from causes other than stroke).^29^

LASSO variable selection and Fine-Gray analyses were performed in R V.3.6.1 using the glmnet package V.3.0–2 and riskRegression package version 8 December 2020, respectively.^21 30^ All other statistical analyses were performed using Python V.3.7.0. Cox proportional hazards models were implemented using the lifelines package version 0.21.1.^31^ AUC analyses were performed using the scikit-learn toolkit V.0.19.2.^32^ Additional details of the methods used for the statistical analyses are provided in online supplemental eMethods IV.

## Results

Among the 503 842 CKB study participants in the present analyses, the mean (SD) age was 51.9 (10.6) years and 59% were women. During 9 years of follow-up, a total of 43 234 individuals had a first incident stroke irrespective of type (total stroke); 36 310 had a first IS and 8865 had a first HS (table 1). The incidence of first total stroke was higher in men than in women (9.5% vs 7.9%) and varied over fivefold across the 10 study areas. Compared with those who had no stroke, individuals who had a first stroke were older and more likely to have prior history of CHD, diabetes or hypertension. Individuals who had HS were more likely to be current smokers and have higher mean levels of systolic blood pressure than those who had IS. Overall, men and women had similar rates of prior history of CHD (2.5% vs 3.0%), diabetes (5.3% vs 6.0%), and use of blood pressure-lowering medication (9.9% vs 11.4%), but current smoking was much more common in men than in women (67.7% vs 3.2%) (table 1).

**Table 1:** Distribution of established risk factors for total stroke and stroke pathological types in men and women in CKB

Risk factors included in FSRP*	Men				Women			
	No stroke (n=185 706)	Total stroke (n=19 587)	IS (n=16 113)	HS (n=4587)	No stroke (n=274 902)	Total stroke (n=23 647)	IS (n=20 197)	HS (n=4278)
Age, mean, year	51.8	60.7	60.8	60.8	50.6	59.6	59.7	59.4
Current smoking, %	68.5	59.9	58.7	63.8	3.1	4.8	4.7	5.4
Coronary heart disease, %	2.1	6.4	6.8	4.8	2.5	9.4	10.0	5.6
Age 65 years+, %	13.8	39.9	39.9	41.4	10.7	34.0	34.1	34.6
Diabetes at age <65 years, %	3.7	6.5	7.0	4.7	4.0	8.1	8.6	6.3
Diabetes at age 65+ years %	1.1	4.8	5.3	3.8	1.3	6.0	6.3	4.7
BP-lowering treatment, %	8.6	22.3	22.6	22.9	10.1	26.3	26.3	29.4
SBP-untreated, mean, mm Hg	130	142	141	148	126	138	137	148
SBP-treated, mean, mm Hg	148	153	152	158	150	155	154	162

‘No stroke’ column includes individuals lost to follow-up before 9 years, and were stroke-free until being censored.

*Atrial fibrillation was not recorded in CKB.

CKB, China Kadoorie Biobank; FSRP, Framingham Stroke Risk Profile; HS, haemorrhagic stroke; IS, ischaemic stroke; SBP, systolic blood pressure.

### Assessment and update of FSRP for prediction of total stroke

**Figure 1:**
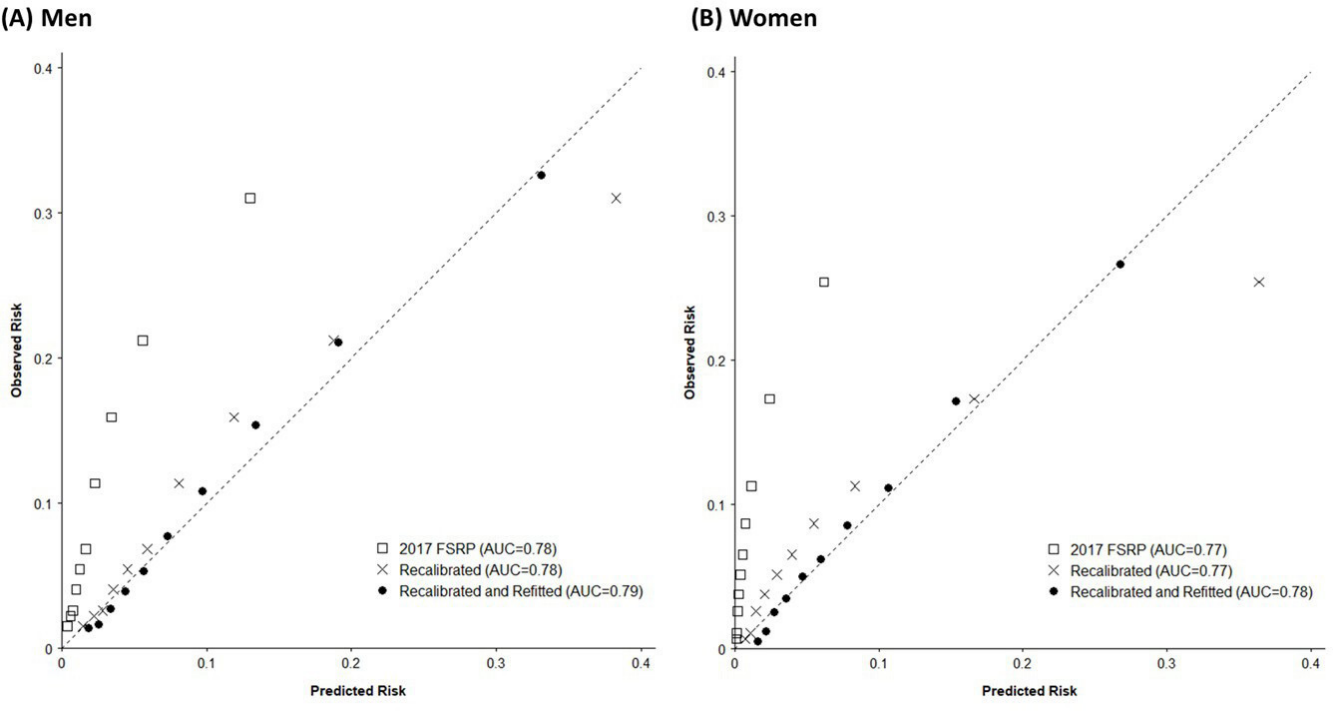
Calibration of the 2017 FSRP, and recalibrated and refitted models from China Kadoorie Biobank, for total stroke in men and women. The dashed line in each subplot represents the line of equality between observed risk and predicted risk. Models with better calibration have points lying closer to the line of equality. Observed 9-year incidence calculated as 1 − Kaplan-Meier estimate. AUC, area under the curve; FSRP, Framingham Stroke Risk Profile.

The 2017 FSRP yielded moderate discrimination for total stroke in CKB (AUC (95% CI): 0.78 (0.77 to 0.79) in men, 0.77 (0.76 to 0.78) in women) (table 2). However, calibration was very poor, and the 2017 FSRP substantially underestimated the absolute risk of total stroke (χ^2^: 1825 in men, 3053 in women) (table 2; figure 1).

**Table 2:** Comparison of performance of different models for prediction of total stroke and stroke pathological types in men and women in China Kadoorie Biobank

	Men			Women		
	Discrimination	ΔAUC	Calibration	Discrimination	ΔAUC	Calibration
	AUC (95% CI)	P value	χ^2^	AUC (95% CI)	P value	χ^2^
**Total stroke**						
2017 FSRP	0.78 (0.77 to 0.79)	–	1825	0.77 (0.76 to 0.78)	–	3053
+Recalibration	0.78 (0.77 to 0.79)	–	156	0.77 (0.76 to 0.78)	–	506
+Refitting	0.79 (0.79 to 0.80)	+0.01 (<0.001)	51	0.78 (0.77 to 0.78)	+0.01 (<0.001)	148
+Area stratification	0.82 (0.82 to 0.83)	+0.04 (<0.001)	124	0.82 (0.82 to 0.83)	+0.05 (<0.001)	178
+Additional risk factors	0.83 (0.82 to 0.84)	+0.05 (<0.001)	101	0.83 (0.82 to 0.84)	+0.06 (<0.001)	177
Ischaemic stroke						
2017 FSRP	0.77 (0.76 to 0.78)	–	1200	0.76 (0.76 to 0.77)	–	2406
+Recalibration	0.77 (0.76 to 0.78)	–	118	0.76 (0.76 to 0.77)	–	479
+Refitting	0.78 (0.78 to 0.79)	+0.01 (<0.001)	21	0.77 (0.76 to 0.78)	+0.01 (<0.001)	74
+Area stratification	0.82 (0.81 to 0.83)	+0.05 (<0.001)	70	0.82 (0.82 to 0.83)	+0.06 (<0.001)	124
+Additional risk factors	0.83 (0.82 to 0.84)	+0.06 (<0.001)	55	0.83 (0.82 to 0.84)	+0.07 (<0.001)	90
Haemorrhagic stroke						
2017 FSRP	0.79 (0.78 to 0.81)	–	136	0.78 (0.76 to 0.80)	–	70
+Recalibration	0.79 (0.78 to 0.81)	–	58	0.78 (0.76 to 0.80)	–	65
+Refitting	0.80 (0.78 to 0.81)	+0.01 (0.007)	23	0.80 (0.78 to 0.82)	+0.02 (<0.001)	33
+Area stratification	0.81 (0.80 to 0.83)	+0.02 (<0.001)	22	0.81 (0.80 to 0.83)	+0.03 (<0.001)	11
+Additional risk factors	0.82 (0.81 to 0.84)	+0.03 (<0.001)	14	0.82 (0.80 to 0.84)	+0.04 (<0.001)	9

ΔAUC values were calculated as changes from the 2017 FSRP. Modifications to models were applied cumulatively. 2017 FSRP: 2017 FSRP. +Recalibration: Baseline hazard functions re-estimated in China Kadoorie Biobank.+Refitting: Model coefficients from FSRP re-estimated in China Kadoorie Biobank.+Area stratification: Stratification by study area and area-specific estimation of baseline hazard functions.+Additional risk factors: Further sociodemographic, health and lifestyle risk factor indicators selected using LASSO regularisation.

AUC, area under the curve; FSRP, Framingham Stroke Risk Profile; LASSO, least absolute shrinkage and selection operator.

Recalibration did not alter the AUCs, but substantially corrected the calibration of the model (χ^2^: 156 in men, 506 in women). Refitting the HRs for the calibrated equations yielded little material improvement in discrimination (AUC: 0.79 (95% CI 0.78 to 0.80) in men, 0.78 (95% CI 0.77 to 0.78) in women), but further improved calibration (χ^2^: 51 in men, 148 in women). Refitted HRs and additional details of these models are provided in online supplemental eWorkbook I.

### Prediction of total stroke after adjusting for areas in China

**Figure 2:**
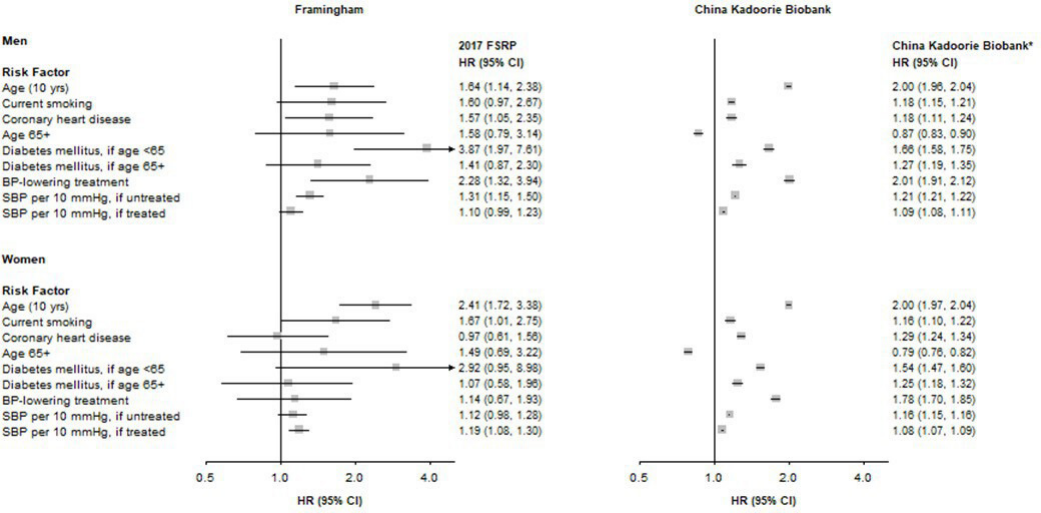
Multivariable HR and 95% CI for total stroke in men and women, for Framingham and for China Kadoorie Biobank. *The 2017 Framingham Stroke Risk Profile (FSRP) coefficients were refitted to China Kadoorie Biobank in a model including stratification by geographical area. BP, blood pressure; SBP, systolic BP.

Stroke incidence rates varied markedly by geographical region within China and online supplememental eFigure 1 demonstrates the baseline survival curves for total stroke, IS and HS for each of the 10 study regions in CKB. Modelling separate area-specific baseline survival functions for total stroke yielded modest, but statistically significant improvement (p<0.001) in risk discrimination among all study participants (AUC: 0.82 (95% CI 0.82 to 0.83) in men; 0.82 (95% CI 0.82 to 0.83) in women), while maintaining good calibration (χ^2^: 124 in men; 178 in women) (table 2). The discrimination performance within each of the 10 areas is reported in online supplemental eTable I. HRs for individual risk factors obtained from these models differed from the 2017 FSRP (figure 2) and demonstrated substantially greater consistency between men and women and had much greater precision (as reflected by the narrower CIs since the CKB population was 100-fold larger than the Framingham cohort). A sensitivity analysis including age at which ever-regular smokers started smoking had larger HRs associated with ever-regular smoking in men (1.37) and women (1.17), but showed no material improvement in risk prediction for stroke (online supplemental eFigure II).

### Expanded risk equations with additional risk factors

In addition to controlling for area-specific differences, the addition of other risk indicators recorded in CKB was assessed for risk prediction of total stroke. The expanded models for total stroke, determined using LASSO regularisation for variable selection, included 66 risk factor indicators for men and 70 in women, including measures of diet, personal and family medical history and socioeconomic status (online supplemental eWorkbook I). These models did not yield any further material improvements in either risk discrimination (AUC: 0.83 (95% CI 0.82 to 0.84) in men, 0.83 (95% CI 0.82 to 0.84) in women) or calibration (χ^2^: 101 in men; 177 in women) (table 2). Discrimination performance within each area is reported in the online supplement (online supplemental eTable II).

### Risk equations for different stroke pathological types

Analysis of separate risk equations for IS and HS demonstrated comparable results from recalibration, refitting, accounting for geographical area, and addition of other risk factors. The best-performing IS model yielded AUCs (95% CI) of 0.83 (0.82 to 0.84) in men and 0.83 (0.82 to 0.84) in women with χ^2^ values of 55 and 90, respectively. The best-performing HS model yielded AUCs of 0.82 (95% CI 0.81 to 0.84) in men and 0.82 (95% CI 0.80 to 0.84) in women with χ^2^ values of 14 and 9, respectively (table 2).

**Figure 3:**
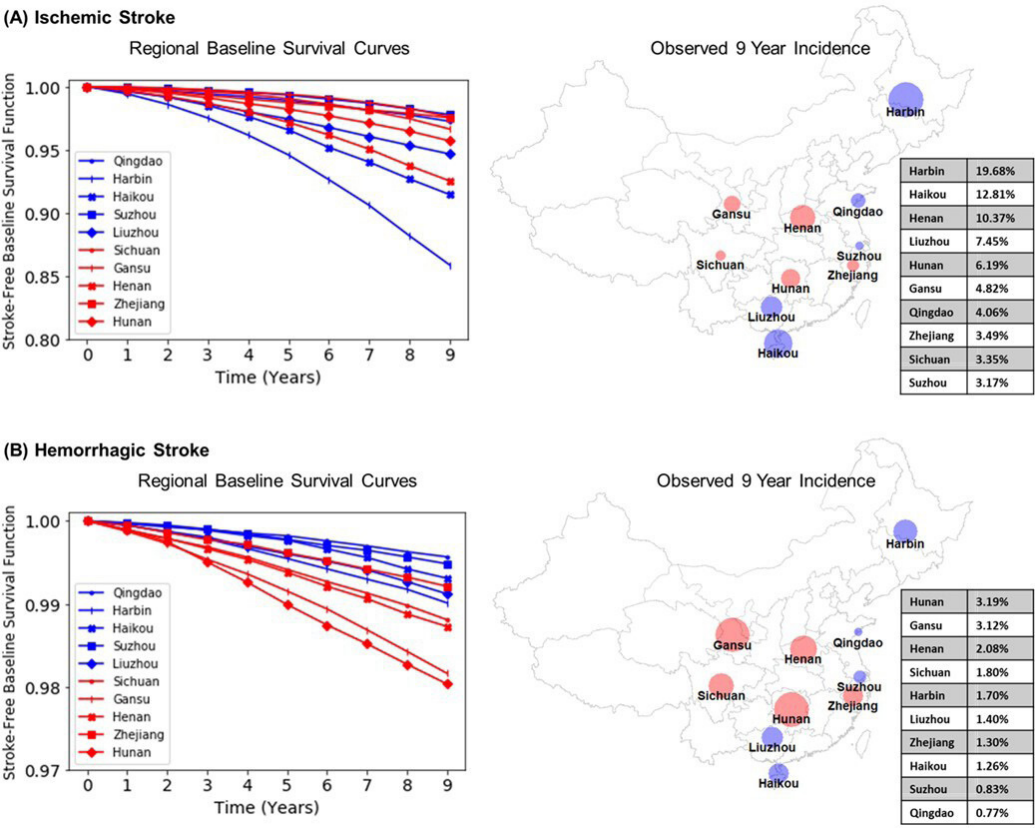
Area-specific baseline survival curves and 9-year incidence for ischaemic stroke and haemorrhagic stroke in China Kadoorie Biobank. Study area-specific baseline survival curves are averaged across sex. Urban study areas are shown in blue while rural areas are shown in red. Note the different scales of the y-axes in subplots (A, B). Dot sizes on maps correspond to observed 9-year incidence (1 − Kaplan-Meier estimate).

The individual risk equations for IS and HS demonstrated substantial differences between the two stroke pathological types. Overall, the absolute risk of IS was 4–5 fold greater than HS, and the ratio of IS to HS risks differed substantially between areas. Modelling area-specific baseline survival curves (ie, predicted survival rates for an individual with mean risk factor values) for IS and HS demonstrated striking differences in stroke risk between areas, consistent with geographical differences in observed stroke incidence during the 9-year follow-up period (figure 3, online supplemental eFigure III). For example, residents in Harbin had threefold higher 9-year incidence of IS compared with those in Hunan (19.68% vs 6.19%), but had half the incidence of HS (1.70% vs 3.19%). Furthermore, by training separate models for IS and HS, different HRs were determined for the same risk factors, including CHD (HR for IS/HS; men: 1.18/1.01, women: 1.32/0.97), diabetes ((age<65 years) men: 1.77/1.44, women: 1.60/1.25; (age 65+years) men: 1.34/1.11, women: 1.30/1.02) and blood pressure-related risk factors (online supplemental eWorkbook I).

### Sensitivity analyses

Restriction to a subset of participants aged ≥55 years yielded comparable results from recalibration, refitting, accounting for area and including additional risk factors (online supplemental eTable III). However, due to increased homogeneity among included individuals, the AUCs were lower after excluding the younger adults (age 30–54 years). The age restriction also yielded more extreme differences in the AUCs for the best-performing models for IS and HS compared with the AUCs for the best-performing models for total stroke. Including additional risk factors prior to area-stratification demonstrated that extra risk factors improved AUCs for total stroke, IS and HS, with area-stratification contributing to further improved discrimination for total stroke and IS only (online supplemental eTable IV). After adjusting for competing causes of death, the incidence rates for total stroke and stroke pathological types were similar to the Kaplan-Meier derived estimates (figure 3, online supplemental eFigure IV), and likewise, the predicted risks from the Fine-Gray and Cox models were also similar (online supplemental eFigure V).

### Opportunities for validation in independent populations

A risk calculator is provided in online supplemetnal eWorkbook I to enable validation of the CKB risk scores for total stroke and stroke pathological types in independent local populations. Details of the methods on how to use the risk calculators are provided in online supplemental eMethods V.

## Discussion

This study, involving a 100-fold larger population than the original Framingham Study, demonstrated that the 2017 FSRP was effective at distinguishing between individuals with and without stroke (good discrimination), but greatly underestimated the absolute risks of total stroke in Chinese adults (poor calibration) due to higher incidence rates of both IS and HS in China compared with Western populations. Absolute risk prediction of total stroke was substantially improved by recalibrating the baseline survival function, with modest additional benefit from refitting HRs (online supplemental eFigure VI). Adjusting for 10 areas in China yielded modest, but statistically significant, improvements in risk discrimination, but there were no further material improvements achieved by adding 38–60 additional risk indicators available in CKB. There was also good performance of separate models for IS and HS, and evidence that the relative importance of predictors differed between these pathological types.

A few population-based prospective studies had previously assessed the utility of FSRP in Chinese adults.^12–14^ Overall, they found modest risk discrimination of FSRP for total stroke, but poor prediction of absolute risks of stroke, consistent with the findings of this study. For example, application of FSRP in the China-PAR study, involving 106 281 adults recruited from 4 cohorts in China with a few thousand recorded stroke events, yielded AUCs of 0.65–0.73, but greatly underestimated absolute risks of total stroke.^12^ These, and other studies, have highlighted the need for recalibration of Framingham-based equations for prediction of cardiovascular disease in LMICs like China.^33 34^ While this study yielded similar findings, it provides several advantages including contemporary risks with much greater precision and reliability due to the very large numbers of well-characterised stroke cases (20-fold greater than the China-PAR study); evaluation of differences by 10 widely distributed geographical areas within China; and separate risk prediction of total stroke, IS and HS.

First, the novel models developed in this study successfully controlled for area-specific differences that were unexplained by analysis of the FSRP risk factors alone. While previous studies such as the China-PAR study have focused on developing a single risk prediction model for the whole country,^12^ the results of this study highlight the importance of tailoring risk predictions for specific areas of China, which have substantial differences in incidence of total stroke. The present report provides novel local models for risk prediction of total stroke in 10 diverse areas of China, which have greater predictive utility than a single nationwide model for clinicians in the individual regions.

Second, the separate analysis by study area and stroke pathological types affords insight into the substantial differences in incidence of IS and HS between different areas within China. Some of this geographical variation may be explained by differences in blood pressure.^35^ However, much of this variation remains unexplained and may possibly reflect differences in detection (eg, from greater use of brain imaging in certain areas). Inclusion of additional risk factors (eg, sociodemographic factors, alcohol) captured most of the geographical variation in HS risk, but only a fraction for IS risk. Consequently, this study suggests that in studies where explicitly controlling for geographical areas is not feasible, the inclusion of additional risk factors in addition to those included in FSRP could capture some of the regional differences.

Third, this study has significant implications for prevention of different stroke pathological types. Current guidelines in both high-income countries and LMICs advocate the use of blood pressure-lowering medication, lipid-lowering medication and antiplatelet treatment for cardiovascular disease prevention.^36–38^ However, individual subtypes of stroke are heterogeneous in their aetiology, and likewise, risk factors have heterogeneous effects on individual stroke types.^15 18 23–25^ This study adds to the available evidence by highlighting differences in the HRs for risk factors such as CHD, diabetes and blood pressure-related variables for IS and for HS, and suggests that evaluating an individual’s risk for separate stroke types (as opposed to total stroke only) may also be informative for primary prevention.

This study also had some limitations. First, AF was not recorded in CKB, so could not be included in the models. However, other population-based studies of comparable age groups in China indicated that the prevalence of AF was substantially lower in China than in Framingham (0.4%–1.7% vs 5.0%),^39–41^ and in 2012, the AF-related stroke prevalence in China was estimated to be 0.13 per 1000 people.^40^ Consequently, although AF is a strong predictor of stroke,^39^ it is likely to affect risk prediction for only a small number of individuals in CKB from 2004 to 2008. As prevalence of AF increases in China,^40 41^ it may be increasingly important to incorporate AF into future local stroke risk equations. Another limitation of CKB was that recorded stroke events were limited to hospitalised strokes (92% having brain imaging to support diagnosis) and death from stroke.^18^ In addition, while the risk equations presented in this study are useful for risk prediction, the HRs for individual risk factors cannot be interpreted causally.

Moreover, the Cox models presented do not account for competing risks of death due to other causes, which may affect risk estimates, particularly in older individuals. However, with low rates of censoring in CKB (5.4%, with 4.8% of censoring due to death), the effect of this limitation is small, as indicated by the comparable 9-year stroke incidence rates of stroke after adjusting for competing risk of death and the similar predicted risks between the Cox and Fine-Gray models.

Finally, the risk equations outlined in the present report were not designed for immediate implementation in clinical practice, which would require additional validation in independent populations in China and potentially other LMICs. To our knowledge, there are currently no contemporary regional cohorts of middle-aged and older adults with sufficiently large sample size in China to perform an external validation. As such datasets become available (eg, via the China Precision Medicine Initiative and establishment of regional electronic health records), future studies can use the calculator provided (online supplemental eWorkbook I) to validate these equations.

## Conclusions

This study developed novel local risk equations for total stroke, IS and HS and demonstrated modest, but statistically significant, improvements over the widely used 2017 FSRP in Chinese adults. Improvements in stroke risk prediction can be attributed to recalibration of baseline survival, refitting HRs and accounting for geographical differences in stroke incidence in China. The addition of a large number of other risk factors yielded no further material improvements, but may be useful in other studies when area-specific differences are not readily estimated. These techniques can be implemented to improve risk prediction in any Chinese or similar populations with unique and geographically diverse risk profiles for stroke. Moreover, separate risk equations for IS and HS could help to identify individuals at high risk of a particular stroke pathological type and guide treatment decisions for primary prevention. These equations should be validated and refined in independent populations before implementing them for prediction of stroke risk in clinical practice.

## Data Availability

Data are available on reasonable request. Access details to a stroke risk calculator are provided in a workbook in the online supplemental materials to enable researchers to calculate risk scores for stroke using their own data. Researchers who are interested in obtaining the raw data from the China Kadoorie Biobank study that underlines this paper should contact ckbaccess@ndph.ox.ac.uk. A research proposal will be requested to ensure that any analysis is performed by bona fide researchers and - where data is not currently available to open access researchers - is restricted to the topic covered in this paper.
