# Supplementary material for: Development, validation and comparison of multivariable risk scores for prediction of total stroke and stroke types in Chinese adults: a prospective study of 0.5 million adults
Source: Stroke Vasc Neurol. 2022 Mar 15;7(4):328–36. doi: 10.1136/svn-2021-001251 (PMC9453839; doi:10.1136/svn-2021-001251)
Supplement: Supplementary data [file svn-2021-001251supp001.pdf]

ONLINE SUPPLEMENTAL FILE 1

Risk scores for prediction of total stroke and stroke types in Chinese adults: a prospective study of 0.5 million adults

Supplementary Methods

eMethods I. Members of the China Kadoorie Biobank Collaborative Group

eMethods II. Definitions of Stroke Types

eMethods III. Testing of Proportional Hazards Assumption

eMethods IV. Model Development and Statistical Analyses

eMethods V. Procedures for Applying Reported Models to Predict 9-Year Risk of First Total Stroke, First Ischemic Stroke, or First Hemorrhagic Stroke.

Supplementary Figures

eFigure I. Area-specific and Aggregate CKB Baseline Survival Curves for First Total Stroke, First Ischemic Stroke, and First Hemorrhagic Stroke in Men and Women

eFigure II. Multivariable Hazard Ratios (HR) and 95% CI for Total Stroke using Framingham (“2017 FSRP” Models) versus CKB (“+ Area Stratification” Models [Modified to Include Ever-Regular Smoking and Age Started Smoking]) Coefficients in Men and Women

eFigure III. Predicted Risk Distributions for (A) Total Stroke, (B) Ischemic Stroke, and (C) Hemorrhagic Stroke by CKB Area.

eFigure IV. 9 Year Incidence for (A) Ischemic Stroke and (B) Hemorrhagic Stroke by CKB Area (Adjusted for Competing Risk of Death).

eFigure V. Comparison of 9-Year Risk Predictions in Test Set for Cox Model (“+Refitting”) and Fine-Gray Model for Total Stroke, Ischemic Stroke, and Hemorrhagic Stroke in Men and Women.

eFigure VI. Calibration of the 2017 Framingham Stroke Risk Profile before and after recalibration and refitting in the China Kadoorie Biobank.

Supplementary Tables

eTable I. Area-Specific Discrimination Performance for Total Stroke and Stroke Pathological Types in Men and Women Using “+ Area Stratification” Models with FSRP Inputs

Chun M, *et al. Stroke Vasc Neurol* 2022; 7:e001251. doi: 10.1136/svn-2021-001251

eTable II. Area-Specific Discrimination Performance for Total Stroke and Stroke Pathological Types in Men and Women Using "+ Additional Risk Factors" Models

eTable III. Discrimination and Calibration Performance for Total Stroke and Stroke Pathological Types in Men and Women (Age 55-84) Using 2017 Framingham Stroke Risk Profile (FSRP)**eTable IV.** Comparison of Discrimination and Calibration Performance of Different Risk Models for Prediction of Total Stroke and Stroke Pathological Types in Men and Women (With Modified Ordering of Incremental Changes Compared to Corresponding Table 2)

Supplementary Files

Online supplemental file 2. Stroke Risk Calculator (Including All CKB Risk Factors, Selected Risk Factors for Each Stroke Risk Equation, and Associated Hazard Ratios)

eMethods I: Members of the China Kadoorie Biobank Collaborative Group

International Steering Committee: Junshi Chen, Zhengming Chen (PI), Robert Clarke, Rory Collins, Yu Guo, Liming Li (PI), Chen Wang, Jun Lv, Richard Peto, Robin Walters.

International Co-ordinating Centre, Oxford: Daniel Avery, Derrick Bennett, Ruth Boxall, Ka Hung Chan, Yumei Chang, Yiping Chen, Zhengming Chen, Johnathan Clarke; Robert Clarke, Huaidong Du, Zammy Fairhurst-Hunter, Hannah Fry, Simon Gilbert, Alex Hacker, Mike Hill, Michael Holmes, Pek Kei Im, Andri Iona, Maria Kakkoura, Christiana Kartsonaki, Rene Kerosi, Kuang Lin, Mohsen Mazidi, Iona Millwood, Qunhua Nie, Alfred Pozarickij, Paul Ryder, Saredo Said, Sam Sansome, Dan Schmidt, Paul Sherliker, Rajani Sohoni, Becky Stevens, Iain Turnbull, Robin Walters, Lin Wang, Neil Wright, Ling Yang, Xiaoming Yang, Pang Yao.

National Co-ordinating Centre, Beijing: Yu Guo, Xiao Han, Can Hou, Qingmei Xia, Chao Liu, Jun Lv, Pei Pei, Canqing Yu.

10 Regional Co-ordinating Centres: Guangxi Provincial CDC: Naying Chen, Duo Liu, Zhenzhu Tang. Liuzhou CDC: Ningyu Chen, Qilian Jiang, Jian Lan, Mingqiang Li, Yun Liu, Fanwen Meng, Jinhuai Meng, Rong Pan, Yulu Qin, Ping Wang, Sisi Wang, Liuping Wei, Liyuan Zhou. **Gansu** Provincial CDC: Caixia Dong, Pengfei Ge, Xiaolan Ren. Maiji CDC: Zhongxiao Li, Enke Mao, Tao Wang, Hui Zhang, Xi Zhang. **Hainan** Provincial CDC: Jinyan Chen, Ximin Hu, Xiaohuan Wang. Meilan CDC: Zhendong Guo, Huimei Li, Yilei Li, Min Weng, Shukuan Wu. **Heilongjiang** Provincial CDC: Shichun Yan, Mingyuan Zou, Xue Zhou. Nangang CDC: Ziyan Guo, Quan Kang, Yanjie Li, Bo Yu, Qinai Xu. **Henan** Provincial CDC: Liang Chang, Lei Fan, Shixian Feng, Ding Zhang, Gang Zhou. Huixian CDC: Yulian Gao, Tianyou He, Pan He, Chen Hu, Huarong Sun, Xukui Zhang. **Hunan** Provincial CDC: Biyun Chen, Zhongxi Fu, Yuelong Huang, Huilin Liu, Qiaohua Xu, Li Yin. Liuyang CDC: Huajun Long, Xin Xu, Hao Zhang, Libo Zhang. **Jiangsu** Provincial CDC: Jian Su, Ran Tao, Ming Wu, Jie Yang, Jinyi Zhou, Yonglin Zhou. Suzhou CDC: Yihe Hu, Yujie Hua, Jianrong Jin Fang Liu, Jingchao Liu, Yan Lu, Liangcai Ma, Aiyu Tang, Jun Zhang. **Qingdao** Qingdao CDC: Liang Cheng, Ranran Du, Ruqin Gao, Feifei Li, Shanpeng Li, Yongmei Liu, Feng Ning, Zengchang Pang, Xiaohui Sun, Xiaocao Tian, Shaojie Wang, Yaoming Zhai, Hua Zhang, Licang CDC: Wei Hou, Silu Lv, Junzheng Wang. **Sichuan** Provincial CDC: Xiaofang Chen, Xianping Wu, Ningmei Zhang, Weiwei Zhou. Pengzhou CDC: Xiaofang Chen, Jianguo Li, Jiaqiu Liu, Guojin Luo, Qiang Sun, Xunfu Zhong. **Zhejiang** Provincial CDC: Weiwei Gong, Ruying Hu, Hao Wang,Meng Wan, Min Yu. Tongxiang CDC: Lingli Chen, Qijun Gu, Dongxia Pan， Chunmei Wang, Kaixu Xie, Xiaoyi Zhang.

Event Adjudication Clinicians: Beijing Tiantan Hospital,Capital Medical University Shuya Li, Haiqiang Qin, Yongjun Wang, **Peking University People's Hospital** Qiling Chen, Jihua Wang, **The 1st Affiliated Hospital of Harbin Medical University** Xiaojia Sun, Lei Wang, Xun Wang, Liming Zhang, Shanshan Zhou, **The 2nd Affiliated Hospital of Harbin Medical University** Hongyuan Chen, Li Chen, Haiyan Gou, Weizhi Wang, Yanmei Zhu, Yulan Zhu, **The 2nd Hospital of Hebei Medical University** Ning Zhang, **Huashan Hospital** Xin Cheng, Qiang Dong, Yi Dong, Kun Fang, Yiting Mao, **Jinling Hospital** Yu An, Peiling Chen, Yinghua Chen, Zhihong Liu, Lihua Zhang **The People's Hospital of Liaoning Province** Xiaohong Chen, Naixin Jv, Xiaojiu Li, Liyang Liu, Yun Lu, Xiaona Xing, **Qingdao Fuwai Cardiovascular**

Hospital Shihao You, **Shengjing Hospital of China Medical University** Xiaoli Cheng, Chaojun Gua, Jinping Jiang, Jingyi Liu, Shumei Ma, **Shenyang Military General Hospital** Xuefeng Yang, **The First People's Hospital of Shenyang** Xiaomo Du, Jian Xu, Xuecheng Yang, Xiaodi Zhao, **West China Hospital, Sichuan University** Zilong Hao, Ming Liu, Deren Wang, **The Second Affiliated Hospital of Suzhou University** Xiaoting Li, **Suzhou Kowloon Hospital Shanghai Jiao Tong University School of Medicine** Lili Hui, Zhanling Liao, Feng Liu, **Qingdao Fuwai Cardiovascular Hospital** Chunning Feng, Dejiang Ji, Fengxia Qu, Wenwen Yuan, **The First Affiliated Hospital of Zhengzhou University** Xin Fu, **Zhongshan Hospital,** Jing Ding, Peng Du, Lirong Jin, Yueshi Mao, Xin Wang.

eMethods II. Definitions of Stroke Types

Ischemic stroke (ICD-10 code I63), including lacunar infarction and non-lacunar infarction, was defined as a focal neurological dysfunction lasting for more than 24 hours with or without neuroimaging evidence of a cerebral infarct.

Hemorrhagic stroke was defined to include intracerebral hemorrhage (ICD-10 code I61) and subarachnoid hemorrhage (ICD-10 code I60). Intracerebral hemorrhage was defined as neurological dysfunction caused by hemorrhage into the brain parenchyma or the ventricular system, excluding those induced by injury, with or without neuroimaging evidence of brain hemorrhage. Subarachnoid hemorrhage was defined as neurological dysfunction caused by hemorrhage into the subarachnoid space, excluding those induced by injury, with or without neuroimaging evidence of such hemorrhage.

All fatal and non-fatal stroke cases were coded using ICD-10 by trained medical staff, who were blinded to other personal information, with further checking and review conducted centrally by trained medical staff. All hospital-reported cases of first stroke also underwent additional clinical adjudication, involving retrieval and review of original medical records and brain imaging reports by clinical specialists in China using a bespoke web-based system. About 92% of the reported first stroke cases had their diagnosis confirmed by brain imaging (CT or MRI). Radiological reports (but not primary brain images) of reported cases of non-fatal stroke were adjudicated by Chinese neurologists using a bespoke online system.[1]

eMethods III. Testing of Proportional Hazards Assumption

The Proportional Hazards (PH) assumption for the FSRP inputs was checked using the Cox PHFitter.check_assumptions method implemented by the lifelines package[2] version 0.21.1 in Python version 3.7.0. This method performs a statistical test to test for any time-varying coefficients, and provides visual plots of the scaled Schoenfeld residuals presented against four time transformations for any risk factor that violates the PH assumption. In each plot, a fitted lowess is also presented, along with 10 bootstrapped lowess lines. Deviations of the lowess line from a constant value are violations of the PH assumption.

Tests of the PH assumption were performed for each FSRP risk factor and separately by sex for CKB participants in the training set (174,499 men; 253,766 women). In CKB men, anti-hypertensive treatment (p<0.005), systolic blood pressure for individuals without hypertension treatment (p<0.005), and systolic blood pressure for individuals with hypertension treatment (p=0.02) were identified to violate the PH assumption (using a p-value threshold of 0.05). In CKB women, the risk factors identified to violate the PH assumption were age (p<0.005), diabetes if under 65 years (p<0.005), diabetes if 65+ years (p=0.01), anti-hypertensive treatment (p<0.005), systolic blood pressure for individuals without hypertension treatment (p<0.005), and systolic blood pressure for individuals with hypertension treatment (p=0.04).

It is important to note that with a large sample size, such as in CKB, even very small violations of the Proportional Hazards Assumption would test as statistically significant. To observe the impact of this effect, we repeated tests of the PH assumption for a randomly selected 10% of CKB men and women (17,449 men; 25,376 women). In men, only systolic blood pressure for individuals without hypertension treatment (p=0.02) and systolic blood pressure for individuals with hypertension treatment (p=0.01) were still identified to violate the PH assumption. However, even among these risk factors, the Schoenfeld residual plots (below) showed a lowess line with very minor deviation from a constant value.

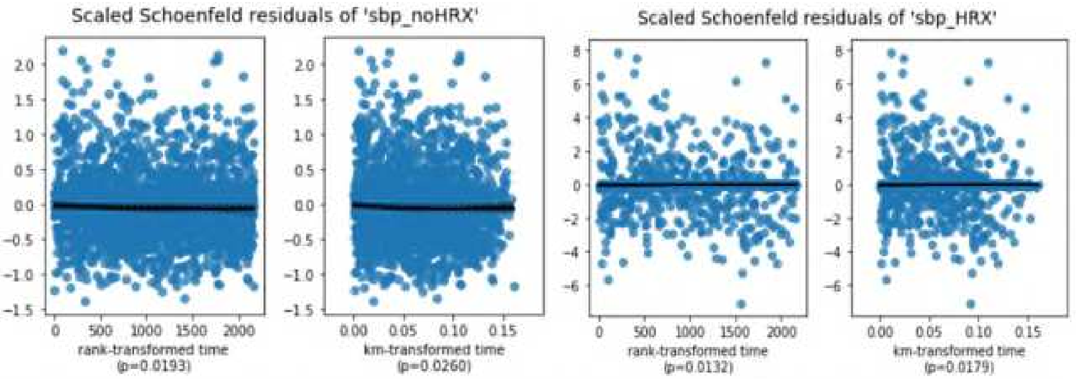

Schoenfeld residual plots for systolic blood pressure for individuals without hypertension treatment (left 2 subplots) and systolic blood pressure for individuals with hypertension treatment (right 2 subplots) among CKB men

In women, only anti-hypertensive treatment (p=0.02) and systolic blood pressure for individuals without hypertension treatment (p=0.03) were still identified to violate the PH assumption. Once again, the Schoenfeld residual plots for these risk factors (below) showed a lowess line with very minor deviation from a constant value. The results of these tests suggest that while the hazards for every FSRP risk factor may not be perfectly proportional, the PH assumption may still be appropriate in this setting.

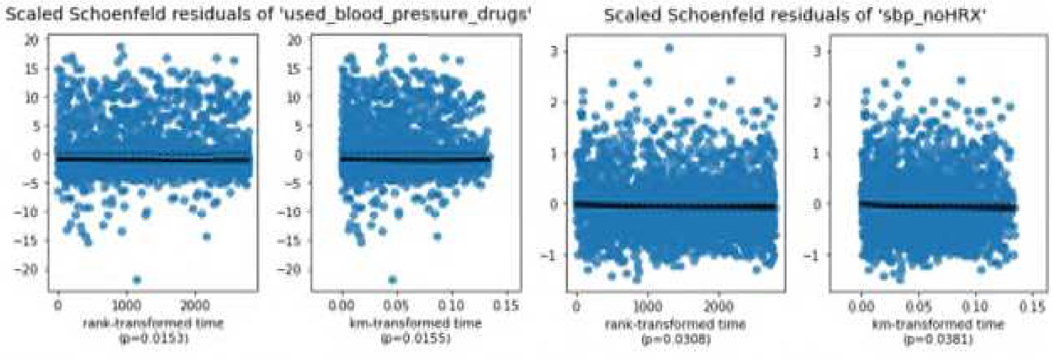

Schoenfeld residual plots for anti-hypertensive treatment (left 2 subplots) and systolic blood pressure for individuals without hypertension treatment (right 2 subplots) among CKB women

Finally, we note that while the PH assumption is important for causal inference, satisfaction of the PH assumption is not necessary for risk prediction applications, where the objectives are optimal risk discrimination and risk calibration irrespective of how individual predictions are generated.

eMethods IV. Model Development and Statistical Analyses

Training Set and Test Set Split

The CKB data was divided into a training set and test set using a random 85%/15% training/test split, stratified by occurrence of the relevant endpoint (i.e., total stroke, IS, or HS) within 9 years of the baseline survey.

Missing Values

Missing values in both the training set and test set were imputed using the means of the non-missing values in the training set. CKB has very few missing values. Of the 133 risk factors considered in CKB (listed with definitions in eWorkbook 1), only 16 risk factors had missing values. Out of all 503,842 individuals included in the present analyses, 521 individuals had missing values for number of siblings and siblings' medical history (stroke, heart attack, diabetes, and cancer); 817 had missing values for mother's medical history (stroke, heart attack, diabetes, and cancer); 1,124 had missing values for father's medical history (stroke, heart attack, diabetes, and cancer); 2 had missing values for weight and BMI; and 228 had missing values for body fat percentage. In addition to mean imputation, 3 binary risk factors were added to represent whether or not an individual was missing medical history for their mother, father, or siblings, respectively. This resulted in a total of 133 potential CKB inputs.

Model Construction

All of the risk equations evaluated in the present analyses were developed using the Cox proportional hazards regression model. Hazard ratios for risk factors were derived from Cox regression while the baseline hazard function (and corresponding baseline survival function) were derived from the non-parametric Breslow estimator.[3,4]

For model construction, recalibration of the baseline survival function and refitting of hazard ratios were conducted in the training set. A summary of the recalibration and refitting methodology is provided in the following diagram, and further details are provided below.

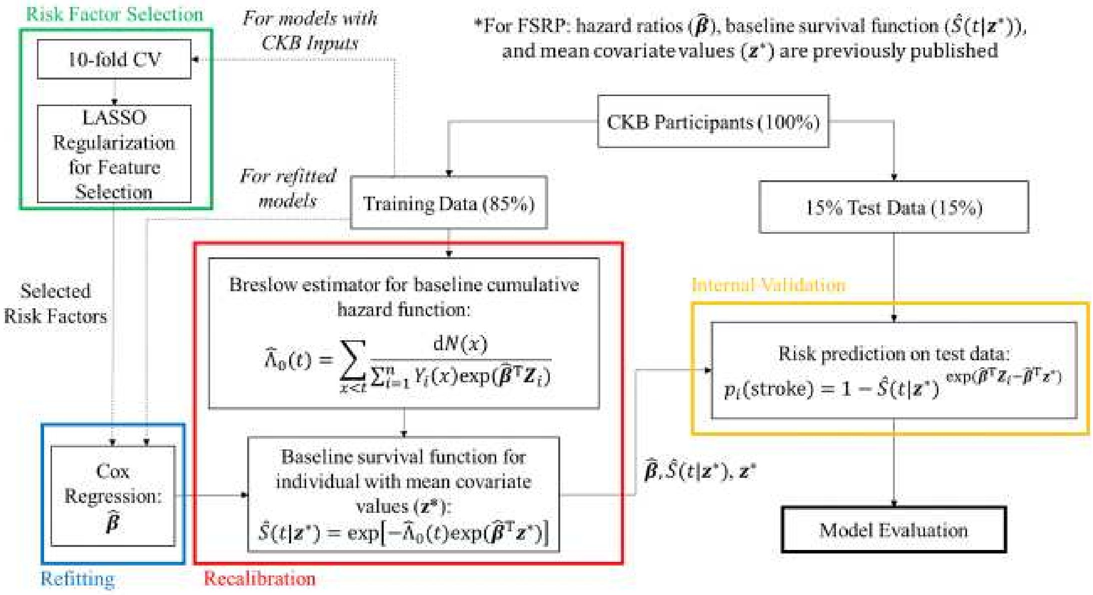

Aggregate Baseline w/ FSRP Inputs: For the following models, a single "aggregate" baseline survival function was implemented for all individuals in the study population. We describe this as an "aggregate" baseline because the survival function was based on a combination of all individuals regardless of their area.

2017 FSRP ("2017 FSRP"): For these models, the published 2017 FSRP baseline survival function and FSRP hazard ratios were used without performing any recalibration or refitting procedures.

Recalibrated FSRP ("+ Recalibration"): For these models, an aggregate CKB baseline survival function was derived. 2017 FSRP hazard ratios were used without refitting.

Recalibrated and Refitted FSRP ("+ Refitting"): For these models, an aggregate CKB baseline survival function was derived and new hazard ratios were refitted for the FSRP risk factors.

Area-specific Baselines w/ FSRP Inputs ("+ Area stratification"): For these models, separate baseline survival functions were developed for each CKB area (10 in total) and new hazard ratios were refitted for the FSRP risk factors using an area-stratified Cox model.

Area-specific Baselines w/ CKB Inputs ("+ Additional risk factors"): For these models, separate baseline survival functions were developed for each CKB area (10 in total). However, rather than limiting the model to FSRP inputs, 10-fold cross-validated LASSO regularization was used (within the training set) for selecting a subset of risk factors from all CKB variables. LASSO regularization was performed separately for each model, yielding slightly different numbers of selected risk factors. The specifics of the variable selection process have been previously described.[5,6] Hazard ratios for the selected risk factors were then fitted using an area-stratified Cox model on the complete training set.

Risk Equations for IS and HS: For these models, identical procedures were performed as described above, replacing total stroke with IS and HS. However, since the FSRP is only provided as a total stroke model, the 2017 FSRP baseline survival function and hazard ratios for total stroke were also used for the IS and HS risk equations.

Model Evaluation

All models were internally validated using the test set. Risk discrimination was evaluated using the area under the receiver operating characteristic curve (AUC), and calibration was evaluated using the Greenwood-Nam-D'Agostino chi-squared statistic ($\chi 2$). Mean values and 95% confidence intervals were determined using 1000 bootstrapped samples from the test set. For comparison, the training set, test set, and bootstrapped samples were designed to be identical for all models predicting the same endpoint (i.e, all total stroke models, all ischemic stroke models, and all hemorrhagic stroke models).

Model Reporting

In order to avoid over-optimism, all reporting about the performance of the risk prediction equations, including AUC and $\chi 2$ metrics, were based on the performance of these models in the test set only.

However, in order to capture information from all CKB individuals in our final models, we report hazard ratios in Figure 2 and eFigure II, risk predictions in eFigure III, and full risk equations in eWorkbook I after reconstructing each model using the overall study population.

eMethods V. Procedures for Applying Reported Models to Predict 9-Year Risk of First Total Stroke, First Ischemic Stroke, or First Hemorrhagic Stroke

Caution: The models presented in this report should not be used in clinical practice before being validated and refined in independent populations. These procedures are included for reporting transparency and reproducibility purposes only.

Calculating a Stroke Risk Prediction for an Individual

We include an interactive tool for exploring the risk equations reported in this study. To determine an individual's 9-yr predicted risk of stroke, refer to the attached "Stroke Risk Calculator" (**Online supplemental file 2**). Using the "Model Selection Options" section in columns A and B, use the provided dropdown menus to select the model of interest.

**Model Selection Options:**	*Use options below to select appropriate stroke risk equation.*
**Select Prediction:**	9Yr Total Stroke Risk
**Select Sex:**	Male
**Select Model:**	FSRP Inputs (Recalibrated - Area-specific CKB Baselines)
**Select Geographic Area:**	Haikou (Urban)
	Haikou (Urban)
	Suzhou (Urban)
	Liuzhou (Urban)
	Sichuan (Rural)
	Gansu (Rural)
	Henan (Rural)
	Zhejiang (Rural)
	Hunan (Rural)

- *Select Prediction*: Allows user to select between "9Yr Total Stroke Risk", "9Yr Ischemic Stroke Risk", and "9Yr Hemorrhagic Stroke Risk"
- *Select Sex*: Allows user to choose between "Male and "Female" options.
- *Select Model*: Allows user to select model-of-interest from the present study
- *Select Geographic Area*: Allows user to select area of individual. If the selected model-of-interest does not require the individual's area, "N/A" will be the only available option.

After completing the "Model Selection Options", refer to columns D and E for the "Individual Risk Factor Values" section. Depending on the selected model, corresponding risk factor prompts will appear in column D. Enter the individual's risk factor values in column E. If any prompts are cut-off, you may need to rewrap the text in the cell. This can be done by highlighting the relevant cell and toggling the "wrap text" button in the "Home" tab of Excel.

**Individual Risk Factor Values:**	
**Risk Factors:**	*Insert risk factor values for individual in this column*
Age (yrs)	40
Current Smoking (0 for no; 1 for yes)	0
History of CHD? (0 for no; 1 for yes)	0
Has Diabetes? (0 for no; 1 for yes)	0
Used BP-lowering drugs? (0 for no; 1 for yes)	0
SBP (in mmHg)	130

Once all risk factor values are entered, a calculated predicted 9-yr risk will be presented in the "Model Output" section in columns G and H.

**Model Output:**	
Predicted 9-yr Risk:	5.41%

<u>Additional Information on Reported Models</u>

The worksheet titled "Model Params - HRs" includes a full listing of all CKB variables, the selected risk factors used in each model (displayed in green), and their corresponding hazard ratios.

The worksheet titled "Model Params - Base Surv" includes the baseline survival function, evaluated at 9 years from time-of-prediction, for each model.

The worksheet titled "Model Params - Means" includes, for all CKB variables, the average risk factor values for the CKB men and women included in the present study. If any risk factor values are unknown for an individual, refer to this worksheet and enter the corresponding mean value into the "Stroke Risk Calculator" worksheet.

The worksheet titled "Calculation" pulls in the appropriate hazard ratios, mean risk factor values, individual risk factor values, and baseline survival function values corresponding to the user's input on the "Stroke Risk Calculator" worksheet. It then walks through the calculations performed by the selected model to generate a risk prediction, which is outputted in cell B16.

The worksheet titled "Risk Factor Definitions" includes the full list of CKB variables and their definitions.

The worksheets titled "Risk Factor Qs" and "Options" are backend sheets used for displaying the appropriate risk factor questions and selection options, respectively, on the interactive "Stroke Risk Calculator" worksheet.

eFigure I. Area-specific and Aggregate CKB Baseline Survival Curves for First Total Stroke, First Ischemic Stroke, and First Hemorrhagic Stroke in Men and Women

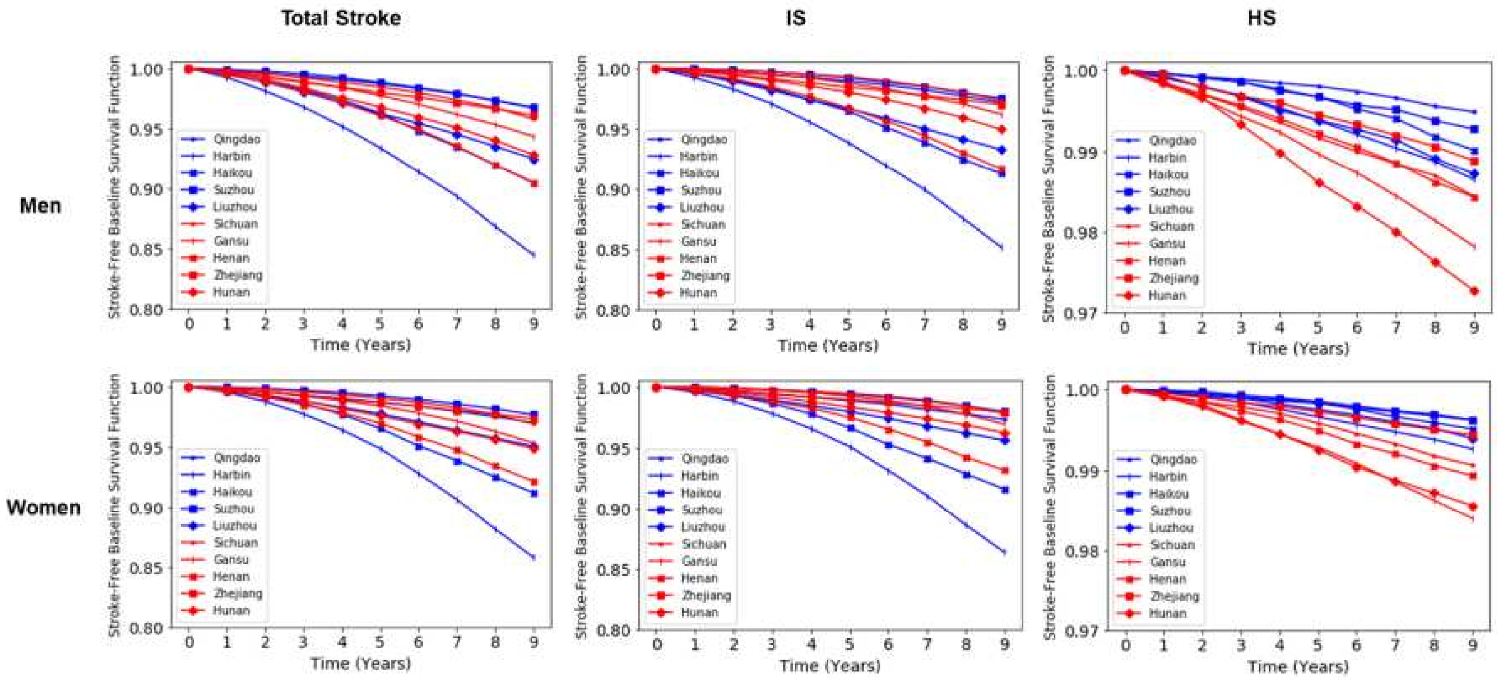

Note: Area-specific baseline survival functions are dependent on the risk factors included in the model as well as their corresponding hazard ratios. In this figure, the area-specific baseline survival functions are shown for recalibrated and refitted models with FSRP inputs only ("+Area stratification" models).

eFigure II. Multivariable Hazard Ratios (HR) and 95% CI for Total Stroke using Framingham ("2017 FSRP" Models) versus CKB ("+ Area Stratification" Models [Modified to Include Ever-Regular Smoking and Age Started Smoking]) Coefficients in Men and Women

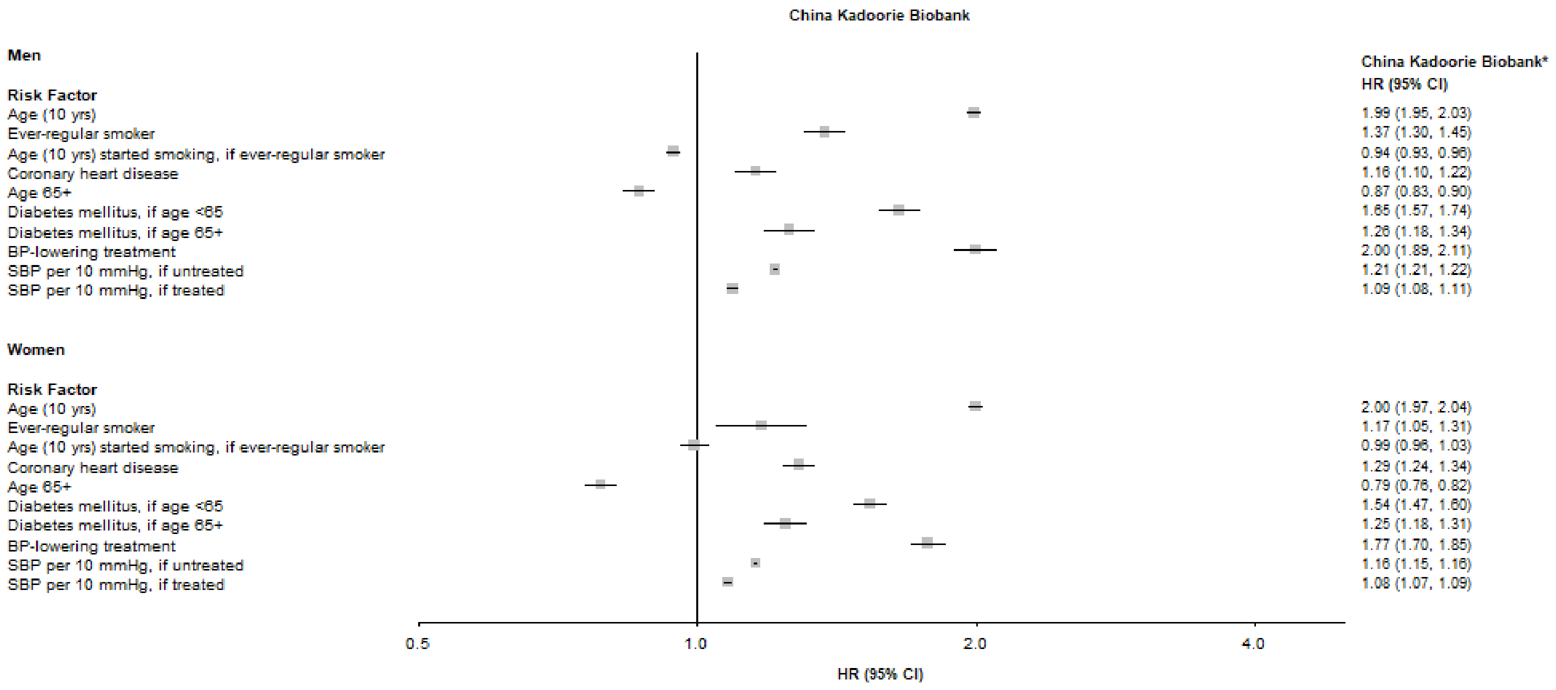

* The 2017 Framingham Stroke Risk Profile coefficients were refitted to China Kadoorie Biobank in a model including stratification by geographical area.

eFigure III. Predicted Risk Distributions for (A) Total Stroke, (B) Ischemic Stroke, and (C) Hemorrhagic Stroke by CKB Area.

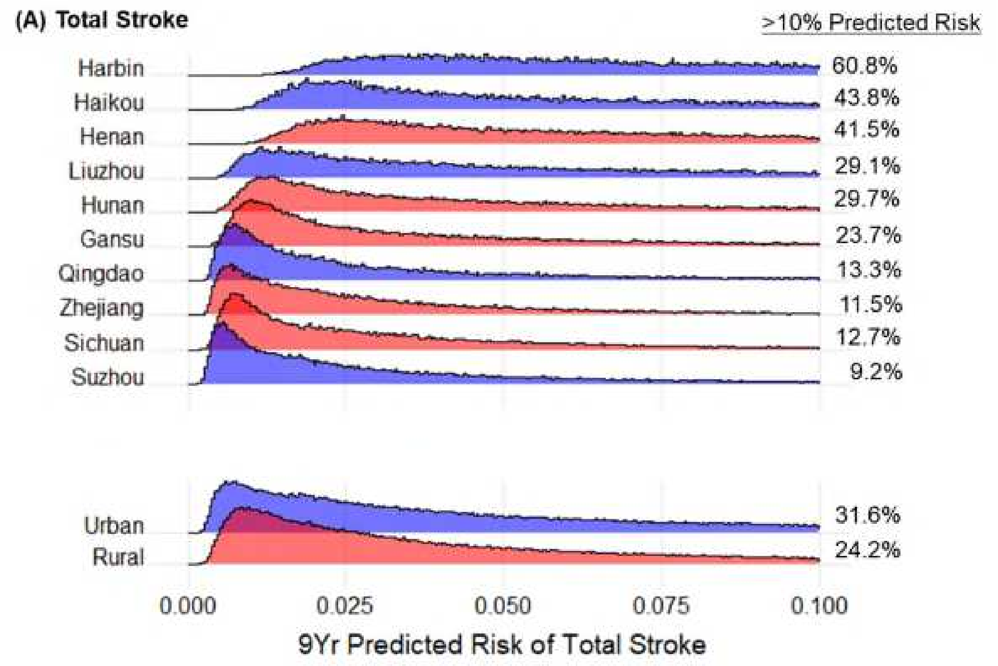

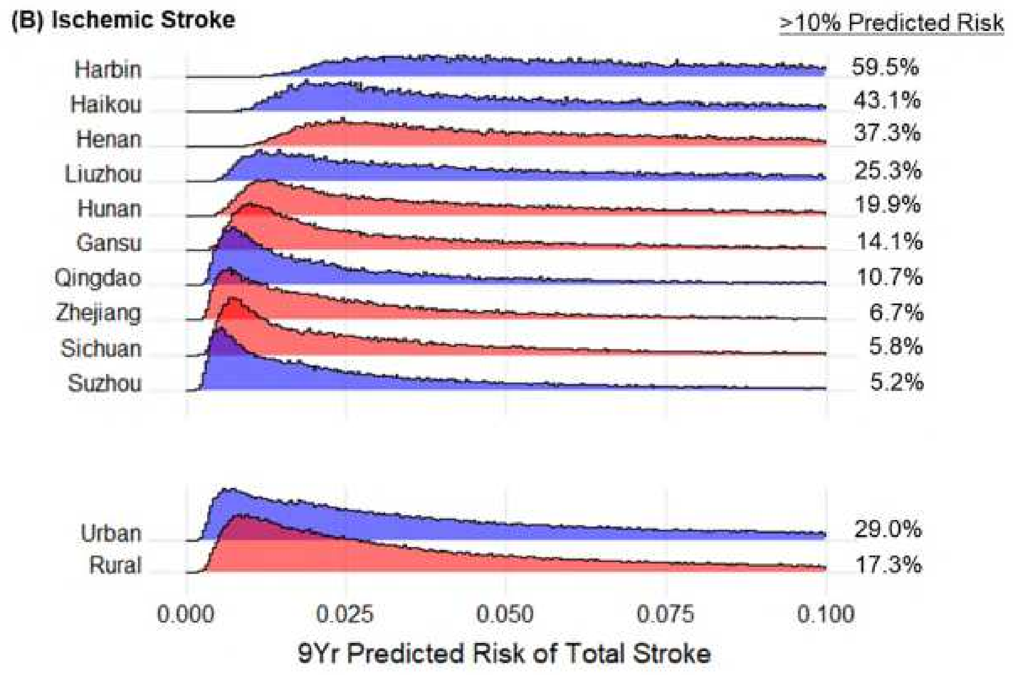

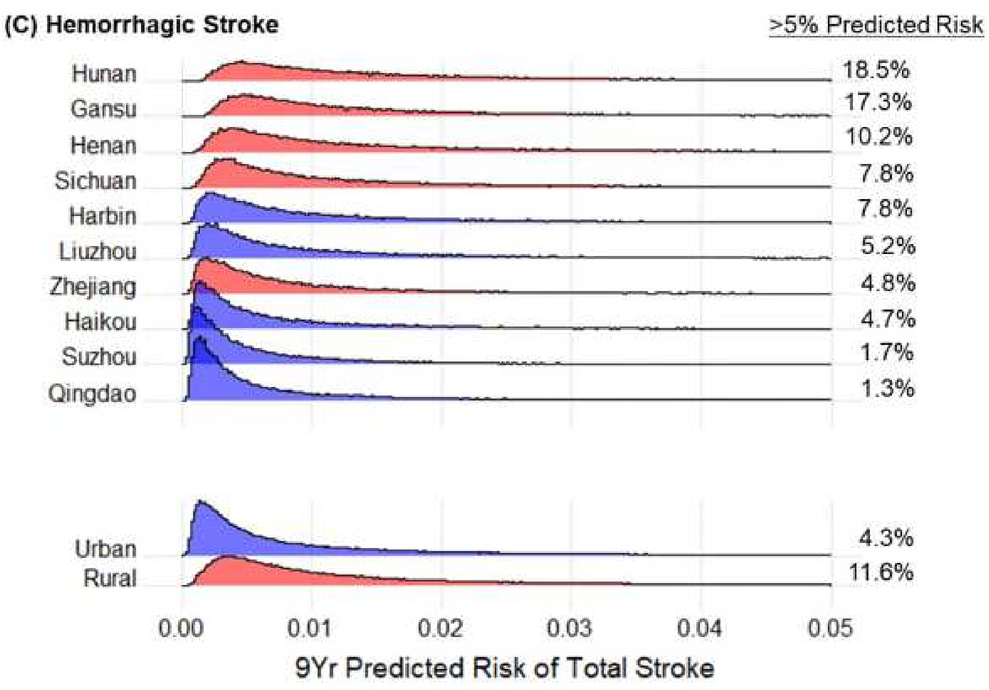

Note: Geographic areas in each subplot are ordered top-to-bottom by decreasing observed 9-year incidence. This corresponds well to the proportion of individuals identified as "high-risk" based on their predicted 9 year risk of stroke (>10% risk for total stroke and IS; >5% risk for HS).

eFigure IV. 9 Year Incidence for (A) Ischemic Stroke and (B) Hemorrhagic Stroke by CKB Area (Adjusted for Competing Risk of Death).

(A) Ischemic Stroke

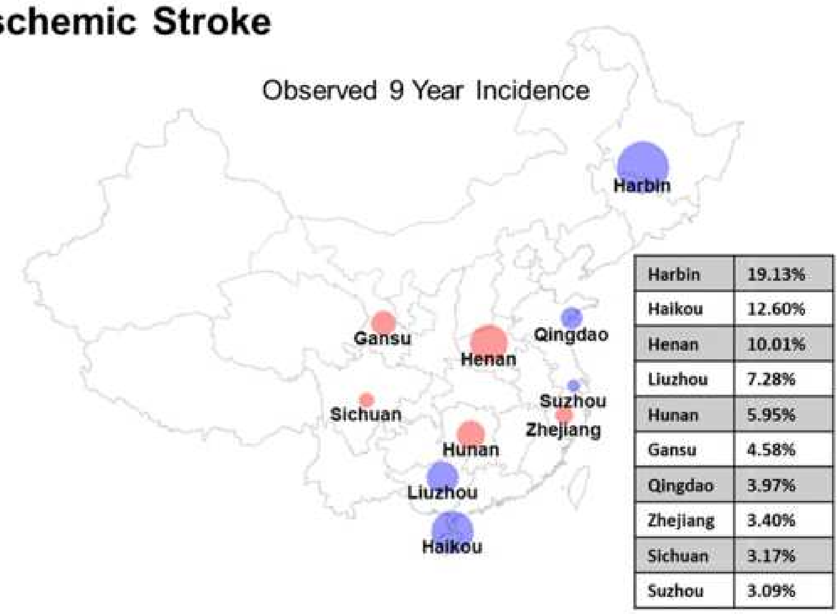

Harbin	19.13%
Haikou	12.60%
Henan	10.01%
Liuzhou	7.28%
Hunan	5.95%
Gansu	4.58%
Qingdao	3.97%
Zhejiang	3.40%
Sichuan	3.17%
Suzhou	3.09%

(B) Hemorrhagic Stroke

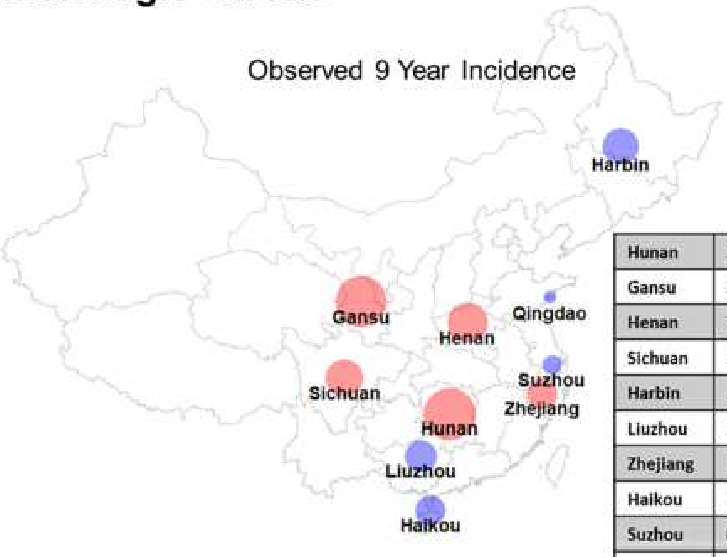

Hunan	3.10%
Gansu	3.02%
Henan	2.01%
Sichuan	1.74%
Harbin	1.65%
Liuzhou	1.36%
Zhejiang	1.26%
Haikou	1.23%
Suzhou	0.81%
Qingdao	0.75%

eFigure V. Comparison of 9-Year Risk Predictions in Test Set for Cox Model (“+Refitting”) and Fine-Gray Model for Total Stroke, Ischemic Stroke, and Hemorrhagic Stroke in Men and Women.

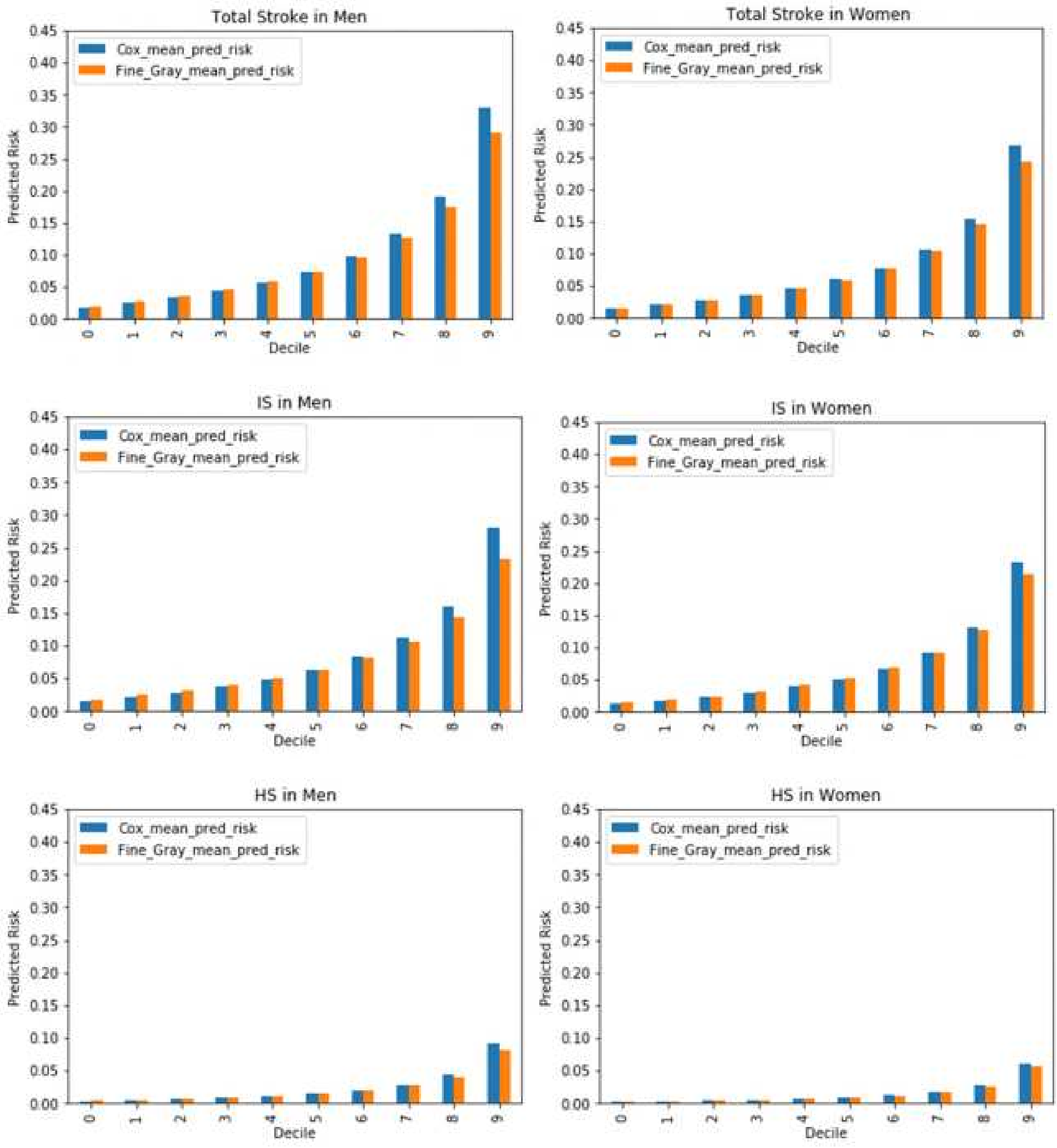

Note: Both Cox and Fine-Gray models were specified with FSRP inputs. Hazard ratios for the Cox models and subdistribution hazard ratios for the Fine-Gray models are reported in eWorkbook 1.

eFigure VI. Calibration of the 2017 Framingham Stroke Risk Profile before and after recalibration and refitting in the China Kadoorie Biobank.

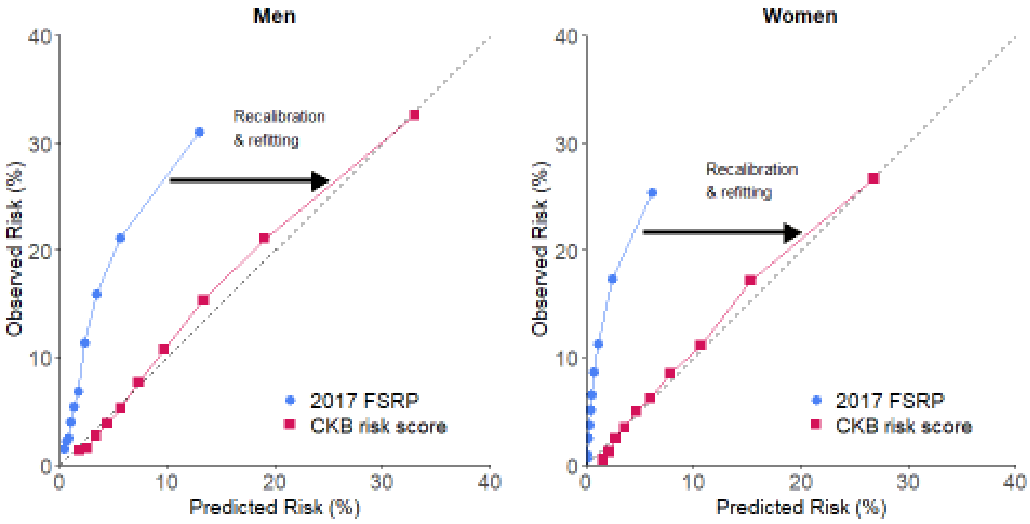

Calibration of the 2017 Framingham Stroke Risk Profile (FSRP) compared to a recalibrated and refitted risk score for prediction of total stroke in the China Kadoorie Biobank (CKB)

eTable I. Area-Specific Discrimination Performance for Total Stroke and Stroke Pathological Types in Men and Women Using "+ Area Stratification" Models with FSRP Inputs

Men

	Total Stroke AUC [95%CI]	IS AUC [95%CI]	HS AUC [95%CI]
Qingdao	0.84 [0.79-0.89]	0.85 [0.81-0.89]	0.81 [0.71-0.90]
Harbin	0.75 [0.73-0.77]	0.75 [0.73-0.77]	0.77 [0.73-0.81]
Haikou	0.80 [0.77-0.83]	0.80 [0.77-0.83]	0.79 [0.70-0.87]
Suzhou	0.82 [0.79-0.85]	0.82 [0.80-0.85]	0.72 [0.62-0.80]
Liuzhou	0.81 [0.79-0.83]	0.77 [0.75-0.80]	0.84 [0.79-0.89]
Sichuan	0.82 [0.79-0.85]	0.82 [0.78-0.86]	0.86 [0.83-0.89]
Gansu	0.80 [0.77-0.83]	0.75 [0.70-0.79]	0.86 [0.82-0.89]
Henan	0.78 [0.75-0.80]	0.77 [0.75-0.79]	0.74 [0.69-0.78]
Zhejiang	0.81 [0.78-0.84]	0.79 [0.75-0.83]	0.79 [0.74-0.83]
Hunan	0.80 [0.78-0.82]	0.78 [0.75-0.80]	0.81 [0.77-0.84]

Women

	Total Stroke AUC [95%CI]	IS AUC [95%CI]	HS AUC [95%CI]
Qingdao	0.81 [0.78-0.84]	0.79 [0.75-0.83]	0.78 [0.71-0.85]
Harbin	0.76 [0.74-0.77]	0.76 [0.74-0.77]	0.80 [0.76-0.84]
Haikou	0.80 [0.77-0.82]	0.77 [0.74-0.79]	0.85 [0.78-0.90]
Suzhou	0.84 [0.81-0.87]	0.82 [0.78-0.85]	0.74 [0.62-0.84]
Liuzhou	0.82 [0.79-0.84]	0.79 [0.77-0.82]	0.75 [0.69-0.81]
Sichuan	0.82 [0.79-0.84]	0.81 [0.78-0.84]	0.86 [0.82-0.90]
Gansu	0.79 [0.77-0.82]	0.77 [0.74-0.80]	0.86 [0.84-0.89]
Henan	0.77 [0.75-0.79]	0.77 [0.75-0.79]	0.77 [0.73-0.81]
Zhejiang	0.82 [0.79-0.85]	0.82 [0.80-0.85]	0.79 [0.73-0.85]
Hunan	0.76 [0.73-0.78]	0.76 [0.73-0.79]	0.79 [0.74-0.83]

Chun M, *et al. Stroke Vasc Neurol* 2022; 7:e001251. doi: 10.1136/svn-2021-001251

eTable II. Area-Specific Discrimination Performance for Total Stroke and Stroke Pathological Types in Men and Women Using "+ Additional Risk Factors" Models

Men

	**Total Stroke** (66 Selected Risk Factors) **AUC [95%CI]**	**IS** (62 Selected Risk Factors) **AUC [95%CI]**	**HS** (42 Selected Risk Factors) **AUC [95%CI]**
Qingdao	0.84 [0.79-0.88]	0.86 [0.81-0.89]	0.84 [0.78-0.90]
Harbin	0.75 [0.73-0.77]	0.77 [0.75-0.79]	0.78 [0.73-0.83]
Haikou	0.79 [0.75-0.82]	0.81 [0.78-0.84]	0.79 [0.70-0.86]
Suzhou	0.83 [0.80-0.86]	0.83 [0.80-0.86]	0.77 [0.67-0.86]
Liuzhou	0.83 [0.80-0.85]	0.78 [0.76-0.81]	0.84 [0.80-0.88]
Sichuan	0.82 [0.79-0.85]	0.82 [0.78-0.86]	0.88 [0.84-0.91]
Gansu	0.81 [0.78-0.83]	0.77 [0.74-0.81]	0.85 [0.82-0.89]
Henan	0.79 [0.77-0.82]	0.77 [0.75-0.79]	0.77 [0.72-0.81]
Zhejiang	0.82 [0.79-0.85]	0.80 [0.76-0.83]	0.79 [0.73-0.84]
Hunan	0.81 [0.79-0.84]	0.79 [0.77-0.82]	0.83 [0.78-0.86]

Women

	**Total Stroke** (70 Selected Risk Factors) **AUC [95%CI]**	**IS** (80 Selected Risk Factors) **AUC [95%CI]**	**HS** (38 Selected Risk Factors) **AUC [95%CI]**
Qingdao	0.82 [0.79-0.85]	0.80 [0.76-0.83]	0.80 [0.73-0.86]
Harbin	0.76 [0.75-0.78]	0.76 [0.75-0.78]	0.81 [0.76-0.86]
Haikou	0.80 [0.78-0.82]	0.77 [0.75-0.79]	0.84 [0.77-0.90]
Suzhou	0.85 [0.82-0.87]	0.82 [0.78-0.86]	0.75 [0.63-0.85]
Liuzhou	0.82 [0.80-0.84]	0.80 [0.77-0.82]	0.74 [0.67-0.81]
Sichuan	0.82 [0.79-0.85]	0.82 [0.79-0.85]	0.86 [0.81-0.90]
Gansu	0.80 [0.77-0.83]	0.78 [0.75-0.81]	0.87 [0.84-0.90]
Henan	0.78 [0.77-0.80]	0.78 [0.76-0.80]	0.79 [0.74-0.83]
Zhejiang	0.83 [0.80-0.86]	0.83 [0.81-0.86]	0.81 [0.75-0.86]
Hunan	0.76 [0.74-0.79]	0.76 [0.74-0.79]	0.79 [0.74-0.83]

eTable III. Discrimination and Calibration Performance for Total Stroke and Stroke Pathological Types in Men and Women (Age 55-84) Using 2017 Framingham Stroke Risk Profile (FSRP)[7]

	Men		Women	
	**Discrimination AUC [95% CI]**	**Calibration χ^2**	**Discrimination AUC [95% CI]**	**Calibration χ^2**
**Total Stroke**				
2017 FSRP	0.68 [0.67-0.70]	1,346	0.66 [0.65-0.68]	2,021
+ Recalibration	0.68 [0.67-0.70]	235	0.66 [0.65-0.68]	248
+ Refitting	0.70 [0.69-0.71]	35	0.68 [0.67-0.69]	22
+ Area stratification	0.73 [0.72-0.74]	27	0.74 [0.73-0.75]	51
+ Additional risk factors	0.74 [0.73-0.75]	34	0.75 [0.74-0.76]	50
**Ischemic Stroke (IS)**				
2017 FSRP	0.68 [0.66-0.69]	928	0.65 [0.64-0.66]	1,588
+ Recalibration	0.68 [0.66-0.69]	209	0.65 [0.64-0.66]	274
+ Refitting	0.69 [0.68-0.71]	35	0.67 [0.66-0.68]	22
+ Area stratification	0.75 [0.74-0.76]	24	0.75 [0.74-0.76]	20
+ Additional risk factors	0.76 [0.74-0.77]	26	0.75 [0.74-0.76]	22
**Hemorrhagic Stroke**				
2017 FSRP	0.71 [0.69-0.74]	93	0.69 [0.67-0.71]	34
+ Recalibration	0.71 [0.69-0.74]	35	0.69 [0.67-0.71]	34
+ Refitting	0.72 [0.70-0.74]	15	0.71 [0.69-0.73]	12
+ Area stratification	0.75 [0.72-0.77]	5	0.75 [0.73-0.77]	28
+ Additional risk factors	0.76 [0.73-0.78]	3	0.77 [0.74-0.79]	11

Note: Atrial fibrillation was not recorded in the CKB.

Chun M, *et al. Stroke Vasc Neurol* 2022; 7:e001251. doi: 10.1136/svn-2021-001251

eTable IV. Comparison of Discrimination and Calibration Performance of Different Risk Models for Prediction of Total Stroke and Stroke Pathological Types in Men and Women (With Modified Ordering of Incremental Changes Compared to Corresponding Table 2)

	Men		Women	
	Discrimination AUC [95% CI]	Calibration χ^2	Discrimination AUC [95% CI]	Calibration χ^2
**Total Stroke**				
2017 FSRP	0.78 [0.77-0.79]	1,825	0.77 [0.76-0.78]	3,053
+ Recalibration	0.78 [0.77-0.79]	156	0.77 [0.76-0.78]	506
+ Refitting	0.79 [0.79-0.80]	51	0.78 [0.77-0.78]	148
+ Additional risk factors	0.81 [0.80-0.81]	84	0.81 [0.80-0.81]	102
+ Area stratification	0.83 [0.82-0.84]	101	0.83 [0.82-0.84]	177
**Ischemic Stroke (IS)**				
2017 FSRP	0.77 [0.76-0.78]	1,200	0.76 [0.76-0.77]	2,406
+ Recalibration	0.77 [0.76-0.78]	118	0.76 [0.76-0.77]	479
+ Refitting	0.78 [0.78-0.79]	21	0.77 [0.76-0.78]	74
+ Additional risk factors	0.81 [0.80-0.81]	28	0.80 [0.79-0.81]	71
+ Area stratification	0.83 [0.82-0.84]	55	0.83 [0.82-0.84]	90
**Hemorrhagic Stroke (HS)**				
2017 FSRP	0.79 [0.78-0.81]	136	0.78 [0.76-0.80]	70
+ Recalibration	0.79 [0.78-0.81]	58	0.78 [0.76-0.80]	65
+ Refitting	0.80 [0.78-0.81]	23	0.80 [0.78-0.82]	33
+ Additional risk factors	0.82 [0.80-0.83]	32	0.82 [0.80-0.83]	6
+ Area stratification	0.82 [0.81-0.84]	14	0.82 [0.80-0.84]	9

Note: Shaded rows indicate changes relative to corresponding Table 2 due to including additional risk factors prior to area stratification.

Chun M, *et al. Stroke Vasc Neurol* 2022; 7:e001251. doi: 10.1136/svn-2021-001251

References

1. Chen Y, Wright N, Guo Y, Turnbull I, Kartsonaki C, Yang L, et al. Mortality and recurrent vascular events after first incident stroke: a 9-year community-based study of 0.5 million Chinese adults. *Lancet Glob Health*. 2020;8:e580-90.

2. Davidson-Pilon C, Kalderstam J, Jacobson N, Reed S, Kuhn B, Zivich P, et al. (2019, April 26). CamDavidsonPilon/lifelines: v0.21.1 (Version v0.21.1). Zenodo. http://doi.org/10.5281/zenodo.2652543
3. Breslow NE. Discussion of the paper by D.R. Cox. *J R Statist Soc B*. 1972;34:215-16.
4. Lin DY. On the Breslow estimator. *Lifetime Data Anal*. 2007;13:471-80.
5. Friedman J, Hastie T, Tibshirani R. Glmnet: lasso and elastic-net regularized generalized linear models. R package version 3.0-2. 2019. http://CRAN.R-project.org/package=glmnet. Accessed April 8, 2020.
6. Hastie T, Qian J. Glmnet vignette. 2014. http://www.web.stanford.edu/~hastie/Papers/Glmnet_Vignette.pdf. Accessed April 8, 2020.
7. Dufouil C, Beiser A, McLure LA, Wolf PA, Tzourio C, Howard VJ, et al. A revised Framingham Stroke Risk Profile to reflect temporal trends. *Circulation*. 2017;135:1145-1159.
8. Wolf PA, D'Agostino RB, Belanger AJ, Kannel WB. Probability of stroke: a risk profile from the Framingham Study. *Stroke*. 1991;22:312-18.
9. D'Agostino RB, Wolf PA, Belanger AJ, Kannel WB. Stroke risk profile: adjustment for antihypertensive medication. The Framingham Study. *Stroke*. 1994;25:40-43.
